# Left Atrial Size and Function in a Canine Model of Chronic Atrial Fibrillation and Heart Failure

**DOI:** 10.1371/journal.pone.0147015

**Published:** 2016-01-15

**Authors:** Adam Goldberg, Kenya Kusunose, Salima Qamruddin, L. Leonardo Rodriguez, Todor N. Mazgalev, Brian P. Griffin, David R. Van Wagoner, Youhua Zhang, Zoran B. Popović

**Affiliations:** 1 Department of Cardiovascular Medicine, Cleveland Clinic, Cleveland, Ohio, United States of America; 2 Department of Molecular Cardiology, Cleveland Clinic, Cleveland, Ohio, United States of America; Loyola University Chicago, UNITED STATES

## Abstract

**Background:**

Our aim was to assess how atrial fibrillation (AF) induction, chronicity, and RR interval irregularity affect left atrial (LA) function and size in the setting of underlying heart failure (HF), and to determine whether AF effects can be mitigated by vagal nerve stimulation (VNS).

**Methods:**

HF was induced by 4-weeks of rapid ventricular pacing in 24 dogs. Subsequently, AF was induced and maintained by atrial pacing at 600 bpm. Dogs were randomized into control (n = 9) and VNS (n = 15) groups. In the VNS group, atrioventricular node fat pad stimulation (310 μs, 20 Hz, 3–7 mA) was delivered continuously for 6 months. LA volume and LA strain data were calculated from bi-weekly echocardiograms.

**Results:**

RR intervals decreased with HF in both groups (p = 0.001), and decreased further during AF in control group (p = 0.014), with a non-significant increase in the VNS group during AF. LA size increased with HF (p<0.0001), with no additional increase during AF. LA strain decreased with HF (p = 0.025) and further decreased after induction of AF (p = 0.0001). LA strain decreased less (p = 0.001) in the VNS than in the control group. Beat-by-beat analysis showed a curvilinear increase of LA strain with longer preceding RR interval, (r = 0.45, p <0.0001) with LA strain 1.1% higher (p = 0.02) in the VNS-treated animals, independent of preceding RR interval duration. The curvilinear relationship between ratio of preceding and pre-preceding RR intervals, and subsequent LA strain was weaker, (r = 0.28, p = 0.001). However, VNS-treated animals again had higher LA strain (by 2.2%, p = 0.002) independently of the ratio of preceding and pre-preceding RR intervals.

**Conclusions:**

In the underlying presence of pacing-induced HF, AF decreased LA strain, with little impact on LA size. LA strain depends on the preceding RR interval duration.

## Introduction

The left atrium (LA) has three sequential roles during the cardiac cycle. First, it acts as a reservoir during ventricular systole, with its compliant chamber stretching to collect incoming pulmonary vein blood while the mitral valve (MV) remains closed. When the MV opens, it acts as a low-resistance conduit for the blood coming from pulmonary veins to fill the left ventricle (LV). Finally, the LA contracts midway through diastole, providing for more efficient LV filling. These three phases interact, as LA contraction leads to a smaller atrium at the closing of the MV, facilitating emptying of the pulmonary venous bed during the reservoir phase, thus decreasing pulmonary vein pressure during LV contraction.

Presence of atrial fibrillation (AF) eliminates atrial “kick” (the coordinated atrial contraction). [1] Loss of atrial kick immediately decreases a systolic component of pulmonary vein flow [1], due to the interaction between LA pump and reservoir functions. Further, during AF the LA does not act as a passive elastic chamber, as AF prevents complete LA relaxation, with electrical and mechanical activity always present in some part of the atrial muscle. Loss of LA compliance can be overcome by increased LA size, and both presence and duration of AF are associated with increased LA volumes. [2] However, little is known about the severity of LA dysfunction and its relevance during AF. Reasons for this include uncertainty about the role RR variability during AF, as well as the lack of agreement on a parameter that adequately reflects LA function in the absence of atrial contraction. Finally, while it appears that vagal nerve stimulation (VNS) improves LA function in HF with sinus rhythm, [3] little is known about the impact of VNS in AF.

Here, using a canine model of HF induced by ventricular tachypacing, we assessed the impact of HF and subsequently induced and maintained persistent AF on LA size and function. Echocardiography was used to assess LA size (volume), and LA function (strain). Our aims were to assess: the impact of AF development on LA size and function; the impact of AF maintenance on LA size and function; the impact of concomitant atrioventricular nodal VNS, and the impact of rate irregularity during AF on LA strain.

## Materials and Methods

### Study Samples

Our experimental design and results related to the impact of AF, HF and VNS on LV function was previously reported. [4] The study was approved by the Cleveland Clinic Institutional Animal Care and Use Committee, and is compliant with the Guide for the Care and Use of Laboratory Animals published by the National Institutes of Health.

Briefly, 24 adult mongrel dogs (21–35 kg; purchased from Fred R Hodgins Kennel, Howell, MI, and Antech Inc, Bamhart, MO) underwent implantation of a modified dual chamber pacemaker (St. Jude Medical, Inc., St. Paul, MN, USA) for induction of HF and subsequent initiation and maintenance of AF. A nerve stimulator (BioControl Medical, Inc., Yehud, Israel) was implanted to deliver VNS. For surgery, all dogs were pre-medicated with thiopental sodium (20 mg/kg i.v.), intubated and mechanically ventilated by a respirator with room air supplemented with oxygen. A surgical plane of anesthesia was maintained using 1–2% isoflurane throughout the experiment. All dogs underwent a right lateral thoracotomy at the 4th intercostal space. Two bipolar epicardial pacing electrodes (Medtronic, Inc., Minneapolis, MN, USA) were sutured to the right atrial appendage and right ventricular apex, respectively, and were connected to the modified dual chamber pacemaker. Another custom-designed bipolar electrode (BioControl Medical, Inc.) was inserted and fixed into the atrioventricular node fat pad, [5] and connected to the nerve stimulator. Both the pacemaker and the nerve stimulator were buried subcutaneously at the back of the dog.

Two weeks after device implant, the ventricular pacemaker was activated in all dogs to induce HF by pacing the ventricles at 220 bpm for 4 weeks. Development of LV dilatation and HF were confirmed by echocardiography.

After induction of HF, ventricular pacing was terminated and the atrial pacemaker was activated (600 bpm) and kept on to induce and maintain AF for the next 6 months. During this time, while the ventricular pacing was stopped, the ongoing AF maintained a rapid ventricular rate. After AF induction, the dogs were randomized in a 2:1 fashion into 2 groups: a control group (n = 9) in which the (HF+AF) condition was maintained, and a vagal nerve stimulation (VNS) group (n = 15) in which atrioventricular node stimulation (0.31 milliseconds, 20 Hz, 3–7 mA) was initiated and continuously delivered to slow ventricular rate to levels corresponding to the spontaneous sinus rate before AF. The nerve stimulator was not activated in the control dogs.

Dogs were monitored daily. Because there are no generally accepted objective criteria for assessing the degree of pain/discomfort in animals, we followed accepted recommendations based on the recognition of a departure from the animal's normal behavior and appearance. [6] Any noticeable behavior changes, including animal's activity, appetite and/or any signs of pain and distress were recorded on the animal's log chart. At the end of the study, 6 months after AF induction, invasive hemodynamic measurements were obtained using micro-transducer tipped catheters, with animals treated per protocol (with or without VNS), and animals were sacrificed.

Cardiac function was assessed biweekly using transthoracic echocardiography. Echocardiographic data were acquired and analyzed by a person blinded to the treatment.

### Data Collection and Analysis

All echocardiographic data acquisition was planned and performed prospectively. Echocardiography was performed using a Vivid 7 echocardiography machine (GE Medical, Milwaukee, WI) at baseline, 4 weeks after the start of HF induction, and every two weeks thereafter. Dogs were trained to lie calmly on their side, and were imaged in left decubitus position while awake. The minimum frame rate acquired during standard 2-dimensional echocardiography was 50 frames/sec. Data were analyzed using EchoPAC PC software (GE Medical, Milwaukee, WI). Maximum LA volumes were measured from the 4- and 2-chamber apical views by the Simpson equation, with special care taken to identify the images with largest LA projection if the animals were in AF.

**Fig 1:**
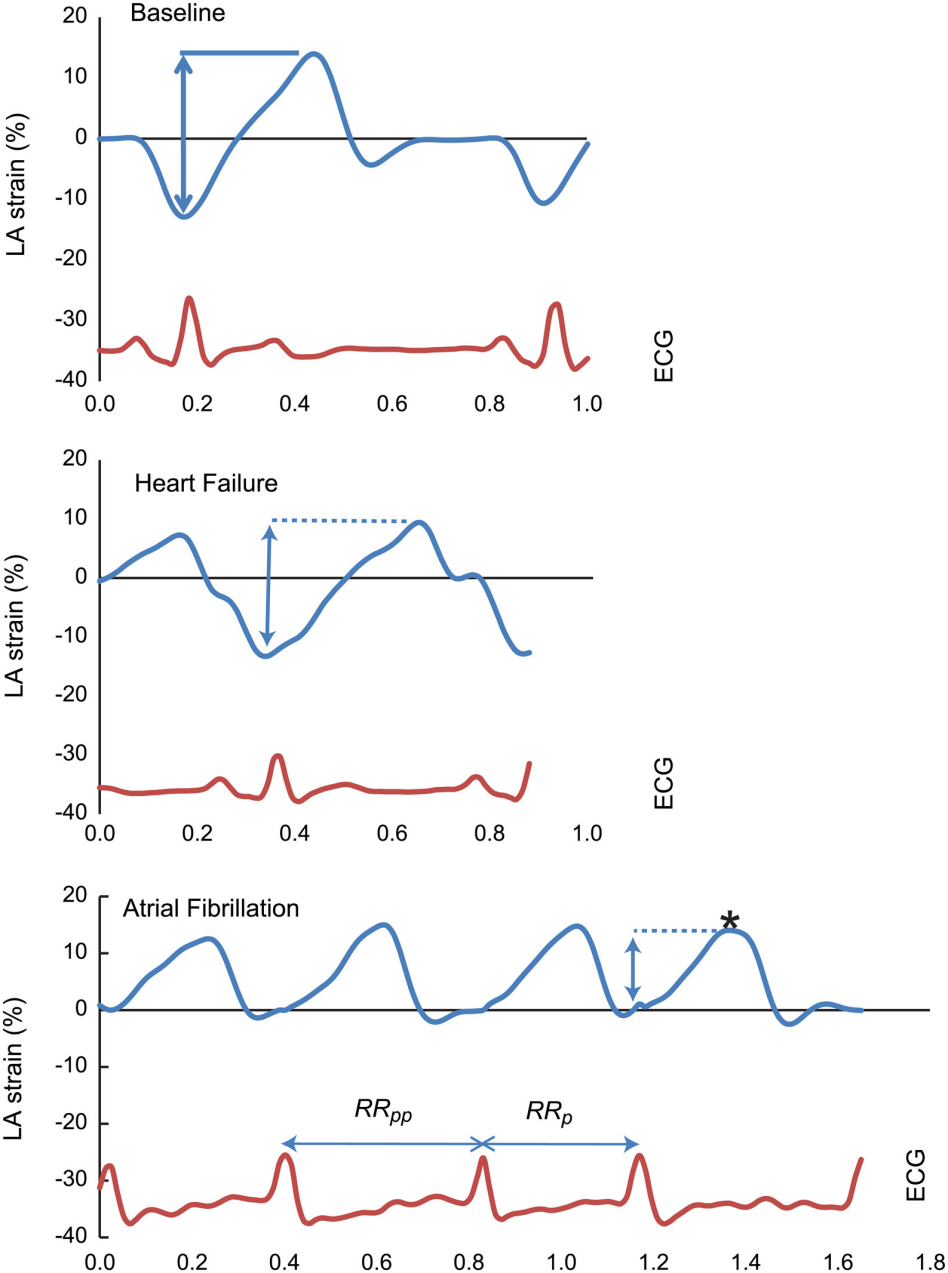
A representative example of left atrial (LA) strain obtained at baseline, after heart failure development with the animal in normal sinus rhythm, and after subsequent atrial fibrilation induction in a same single animal from vagal nerve stimulation (VNS) treated group. ECG: electrocardiogram. RRp and RRpp: RR intervals that precede and pre-precede index left atrial strain (marked by an asterisk). Time (x) axis is in seconds

To extract the LA strain, we used a speckle tracking algorithm incorporated into EchoPAC PC. Again, 4- and 2-chamber apical views were used. After selecting a specific cardiac cycle, the LA endocardial surface was manually traced by a point-and-click approach. From that, a region of interest (ROI) subdivided into six adjoining segments was automatically created, with its width manually adjusted to cover the full thickness of the LA myocardium. Before processing, a cine loop preview was used to confirm that the internal line of the region of interest followed the LA endocardial border throughout the cardiac cycle. The software then generated 6 segmental LA strain curves. After excluding segments with poor tracking (if necessary) the curves were averaged to obtain a single LA strain curve. From that curve, if normal sinus rhythm was present, we used the P-wave as a reference (null) point, with LA strain calculated as the difference between the maximum and minimum of the curve. This value represents total LA strain according to accepted nomenclature.[7] In the presence of AF, the peak of the R wave was used as the reference (null) point, with LA strain calculated as a curve maximum (see Fig 1). We also measured the duration of the RR intervals corresponding to the cardiac cycles in which LA strains were measured. This procedure was repeated for ≥3 cardiac cycles in each of the two views.

The data thus obtained were used in two ways. To assess temporal changes in LA function, average LA strain was calculated by averaging the data from all analyzed individual cardiac cycles obtained in 2- and 4 chamber views. We also used LA strain data of individual cardiac cycles together with the corresponding RR intervals, to assess the impact of RR interval variability on LA strain.

### Statistical Analysis

Continuous data are presented as mean±SD unless otherwise specified. Simple between- group comparisons were performed using the Mann-Whitney U test.

Longitudinal studies in which multiple observations are made through an extended period of time are often unbalanced, and almost always result in missing data due to either technical issues or loss of subjects. In addition, the covariance matrix of the datasets rarely satisfies the criteria of compound symmetry and sphericity needed to perform a standard repeated measures analysis of variance. Because of that, we performed longitudinal data analysis, a statistical method that is applicable to any type of covariance matrix, that can handle subjects with missing data, and can model fixed effects (i.e. effects of type of treatment or time) and random effects (i.e. individual subject characteristics) separately, while being able to handle both linear and non-linear data. [8] The Linear Mixed Effects model that was applied used unstructured covariance for random effects (SPSS statistical software, version 21.0, SPSS Inc. Chicago, IL).[9] To model the impact of HF, AF induction and AF duration, we fitted the data to the equation:

$$B{\;}_{0}+\;B{\;}_{1}\;\times \;HF+\;B{\;}_{2}\;\times \;AF\;+\;B{\;}_{3}\;\times \;VNS+B4\;\times \;time_{AF}+\;B{\;}_{5}\;\times \;AF\;\times \;\;VNS+B6\;\times \;VNS\times time_{AF}\;+\;\;b_{0}\;+\;\;b_{1}\;\times \;HF+\;\;b_{2}\times AF\;+\;\;b_{3}\;\;\times \;\;VNS+\;\;b_{4}\times \;\;time_{AF}$$ (1)

HF, AF, and VNS were coded as 1 if present and 0 if not, while time_AF_ stands for time (in weeks) the animals were in AF. The uppercase letters represent fixed factors, while lowercase letters represent random factors. The fixed parameters were kept if significant (p<0.05) and if the model had lower likelihood ratio and Akaike criterion than otherwise.

To assess the impact of RR interval variability, we performed a nonlinear regression analysis. We first correlated individual, beat by beat data of LA strain obtained in both 4- and 2-chamber views with the duration of preceding RR interval, using the following nonlinear equation, where LA Strain_max_,RR_min_, and time constant are parameters describing the nonlinear relationship:

$$LA\;\;strain=\;LA\;\;strain_{max}\;*\;\;\left(1-e^{\frac{RRmin-preceding\;RR\;interval}{time\;constant}}\right)$$ (2)

Dummy variables were used to code for dog ID, echocardiographic view (2- and 4-chamber) and treatment group. The procedure was repeated while replacing the preceding RR interval with the ratio between preceding and pre-preceding interval (RR_p_/RR_pp_).

The Levenberg-Marquardt method was used to obtain parameter estimates. T-test statistics were used to assess the significance of parameter estimates.

All statistical tests were two- sided, and a P < 0.05 was required for statistical significance.

## Results

As previously reported, [4] one dog in the VNS group was excluded from analysis because it received only intermittent AVN-VNS due to a device technical problem. The remaining 14 dogs in the VNS group successfully completed the study. Of the 9 dogs randomized to the control group, 2 dogs required premature termination of the study due to severe functional deterioration that affected the animals’ welfare, as judged by the institutional veterinarian.

Fig 1 shows a representative example of LA strain obtained at baseline, after HF development (in NSR), and after subsequent AF induction in a single animal from the VNS treated group. Hemodynamic data obtained during the terminal experiments are shown in Table 1. At the time of data acquisition, all dogs were in AF, and all dogs in the VNS group were receiving continuous VNS stimulation. Data were collected from at least 500 consecutive beats. For the calculation of average LV end diastolic and mean diastolic pressures, only beats with LV filling times of >100ms were included.

**Table 1:** Hemodynamic data obtained at sacrifice. All dogs were in atrial fibrillation. In VNS dogs, stimulation continued during data acquisition.

	**LVP_s_ (mm Hg)**	**LVEDP (mmHg)**	**Mean LVDP (mmHg)**	**dLVP/dt_max_ (mmHg/s)**	**dLVP/dt_min_ (mmHg/s)**	**Tau (ms)**
**Controls (n = 6)**	**67±10**	**10±4**	**9±4**	**808±210**	**-963±200**	**96±32**
**VNS (n = 9)**	**78±12**	**9±4**	**7±4**	**1070±286**	**-1313±380**	**81±27**
**p value**	**0.045**	**0.724**	**0.724**	**0.077**	**0.059**	**0.346**

dLVP/dt_max_: maximum left ventricular pressure derivative;

dLVP/dt_min_: minimum left ventricular pressure derivative;

LVP_s_: left ventricular peak systolic pressure;

LVEDP: left ventricular end-diastolic pressure

Mean LVDP: mean left ventricular diastolic pressure;

Tau: time constant of isovolumic pressure decay.

**Fig 2:**
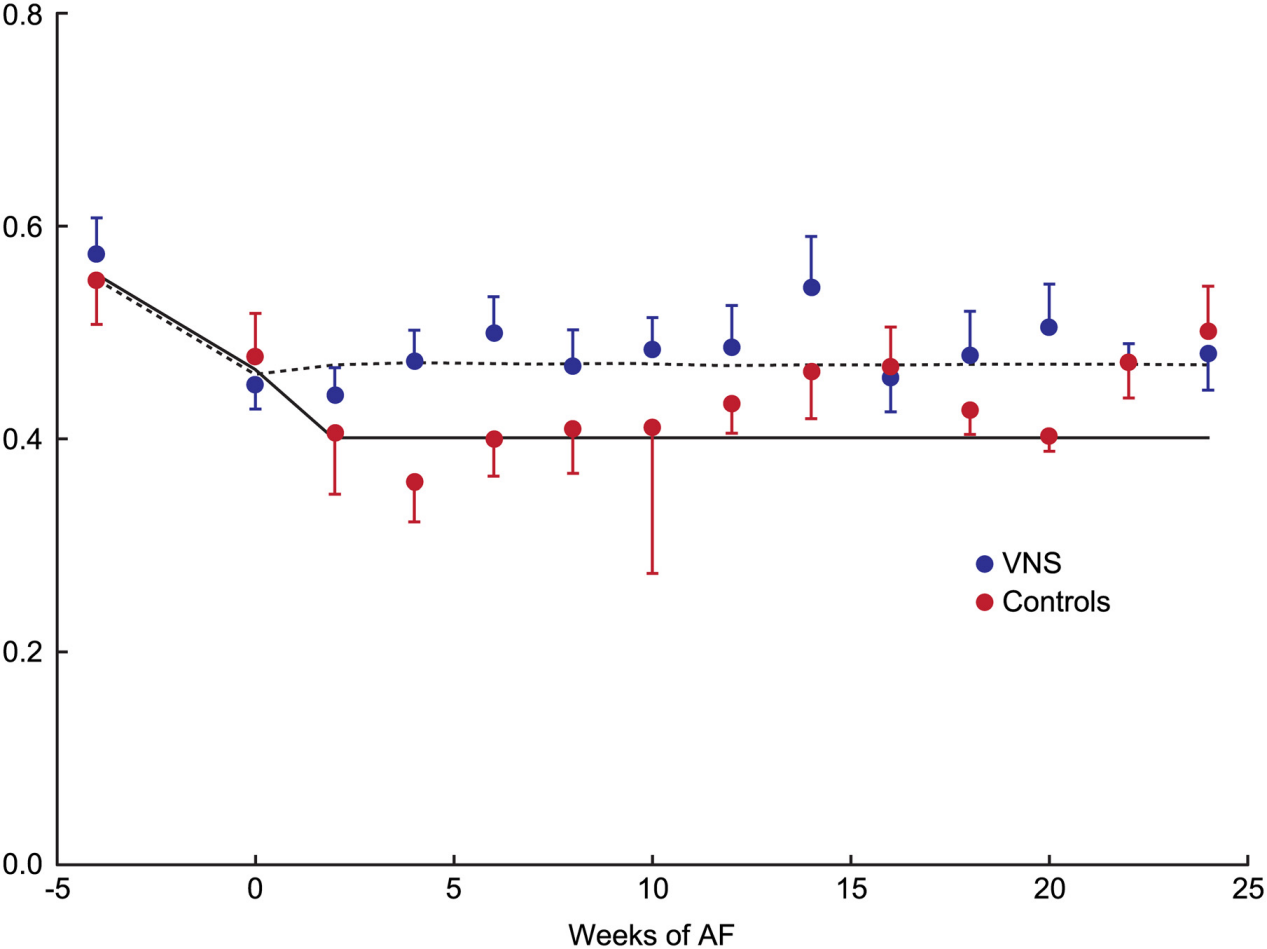
Average RR interval duration in two groups. Error bars represent standard error. The fitted lines are defined by parameter estimates obtained by Linear Mixed Effects model. Please see text for details. AF: atrial fibrillation; VNS: vagal nerve stimulation.

Fig 2 shows average RR interval values corresponding to cardiac cycles in which LA strain was measured throughout the experimental period. RR intervals significantly decreased with development of HF in both group (p = 0.001), and decreased even further with development of AF in the control group (p = 0.014), with a non-significant increase in the VNS group during AF.

**Fig 3:**
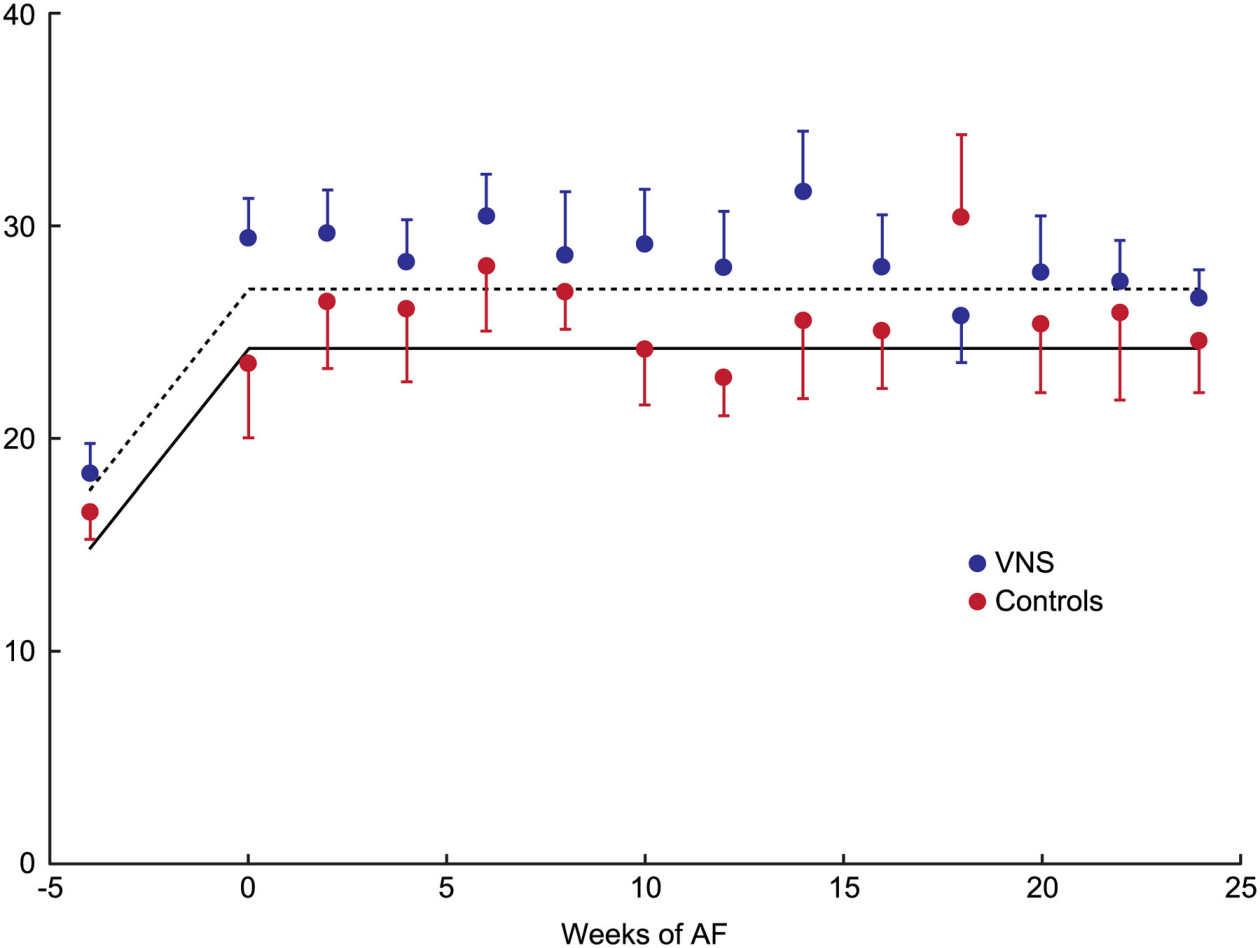
Average left atrial (LA) volume in control and VNS groups. Error bars represent standard error. The fitted lines are defined by parameter estimates obtained by Linear Mixed Effects model. Please see text for details. AF: atrial fibrillation; VNS: vagal nerve stimulation

Fig 3 shows average LA size throughout the experiment. There was a significant increase in LA size during development of HF (P<0.0001), with LA volume nearly doubling. Interestingly, there was no additional increase in LA size during AF in either group, with VNS treated dogs having a trend towards larger LA size throughout the follow-up.

**Fig 4:**
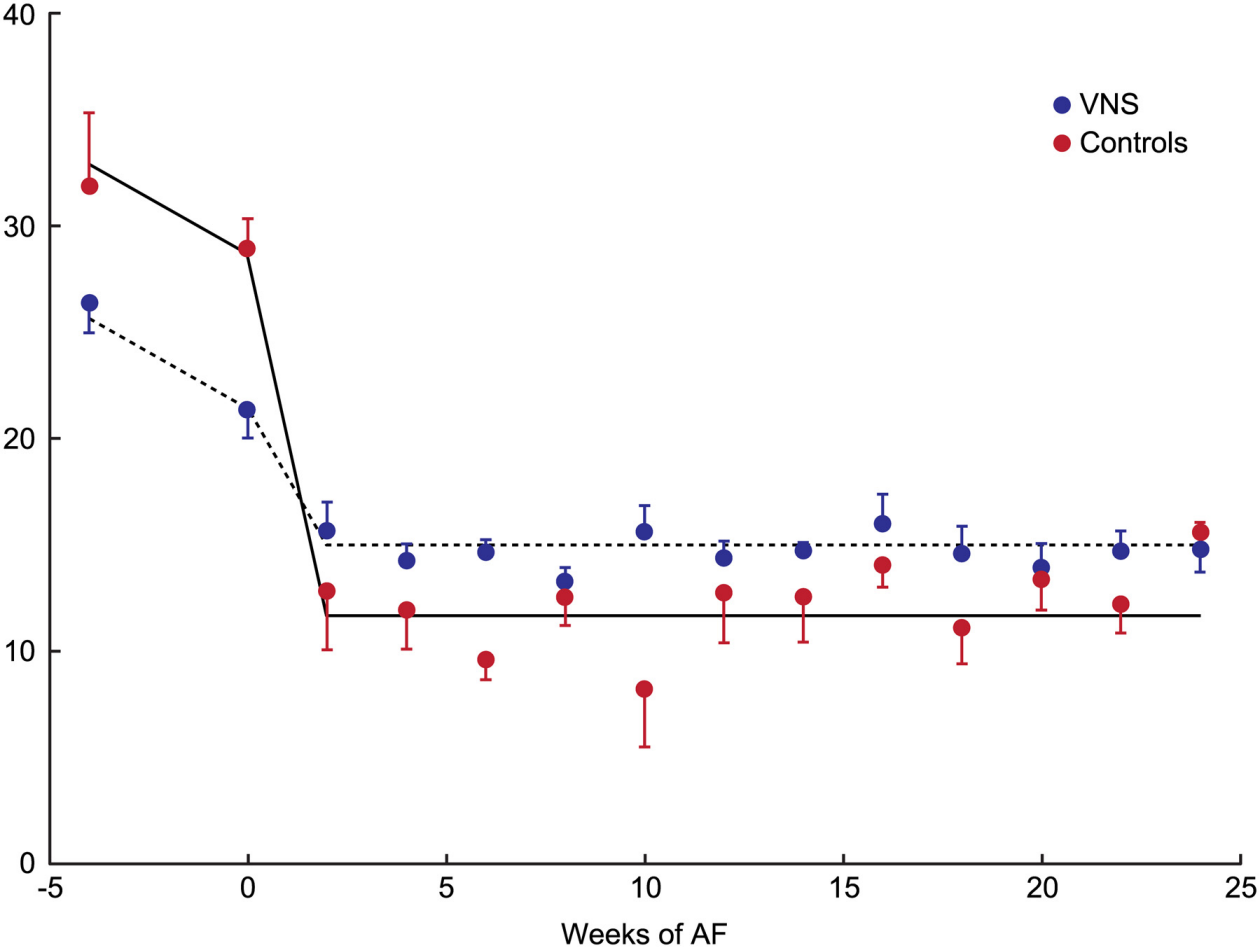
Average left atrial (LA) strain in control and VNS groups. Error bars represent standard error. The fitted lines are defined by parameter estimates obtained by Linear Mixed Effects model. Please see text for details. AF: atrial fibrillation; VNS: vagal nerve stimulation

Fig 4 shows average LA strain throughout the experimental period. LA strain decreased during HF development (p = 0.025). LA strain decreased further after induction of AF (p = 0.0001). However, the reduction of LA strain was significantly smaller (P = 0.001) in the group of AF dogs treated with VNS than in the control (HF + AF) dogs.

Next, we analyzed the impact of RR interval variability on LA strain during AF. Both atrial and ventricular function vary with changes in heart rate and rhythm. However, while multiple studies have analyzed the impact of preceding RR intervals on ventricular function, the impact of variable preceding RR intervals on LA strain is unknown. It might be anticipated that a longer diastolic interval would lead to greater reduction in LA size, while longer subsequent systolic time interval would lead to a larger concomitant increase in LA size, as more time is available for the left atrium to fill. Both intervals are coupled to the duration of the preceding RR interval, with the preceding diastolic interval being part of the preceding RR interval, while the subsequent systolic time interval is dependent on the preceding RR interval duration, due to its impact on both the Starling mechanism and contractility. Thus, we first tested the impact of preceding RR interval duration (RRp) on subsequent LA strain. It can be argued that LA strain is coupled to LV contractility via LV long axis function, and associated mitral annulus descent. Thus, we assessed the impact of RRp/RRpp ratio, a well-known marker for LV contractility in AF [10], on LA strain of a subsequent beat.

**Fig 5:**
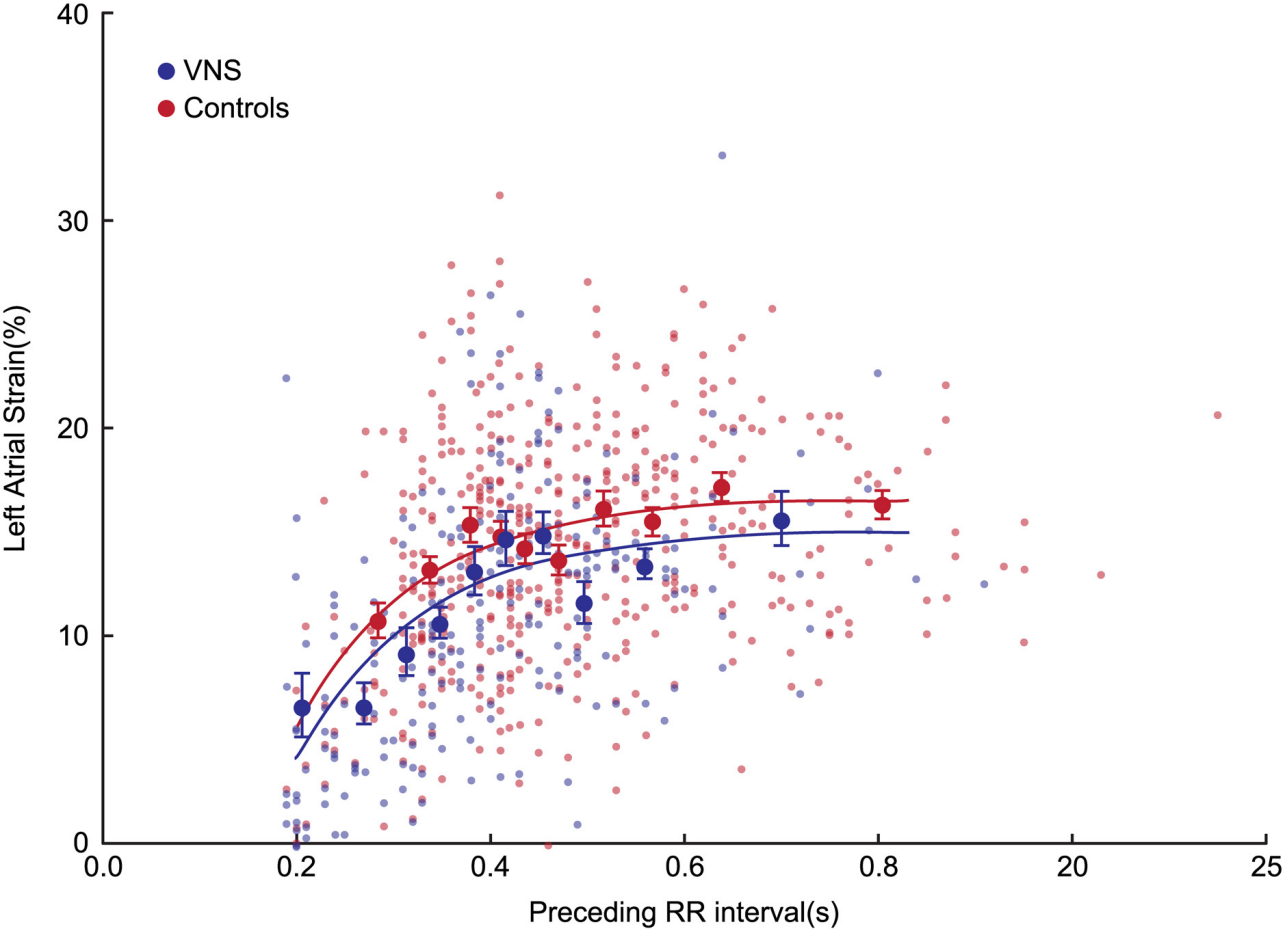
The relationship between preceding RR interval duration [in seconds (s)] and subsequent left atrial (LA) strain in VNS treated and control animals. Left atrial strain data are shown as raw and averaged within deciles or preceding RR duration in each treatment group, with error bars representing standard error. The fitted line was obtained by nonlinear regression. Please see text for details.

**Fig 6:**
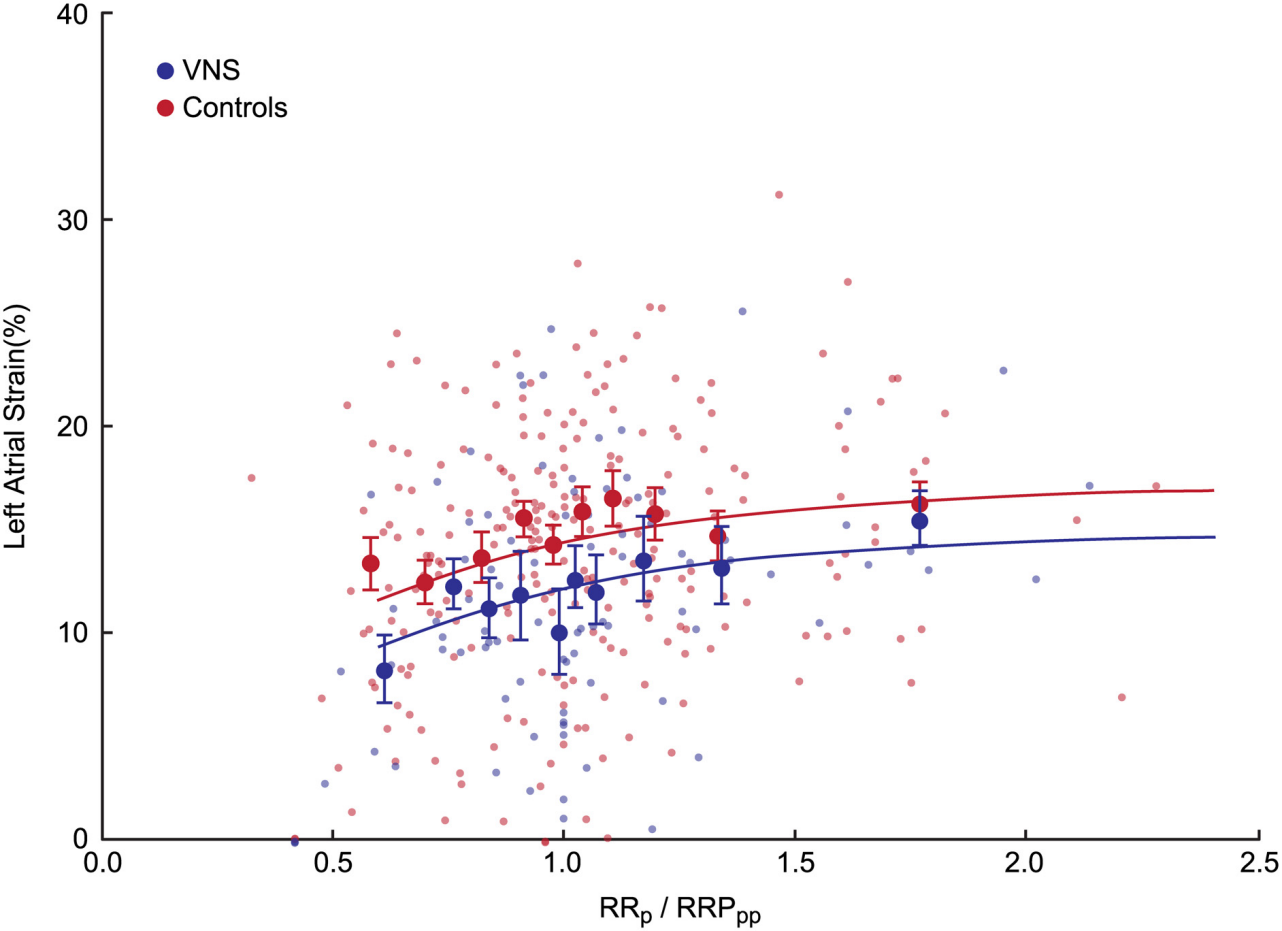
The relationship between the ratio of preceding and pre-preceding RR interval duration (RRp/RRpp) and subsequent left atrial (LA) strain in VNS treated and control animals. . Left atrial strain data are shown as raw and averaged within deciles of RRp/RRpp in each treatment group. The fitted line was obtained by nonlinear regression. Please see text for details.

Fig 5 shows the relationship between preceding RR intervals and subsequent total LA strain. For clarity, both raw LA strain data and LA strain data averaged over deciles of RR interval duration are presented in both treatment groups. The relationship was nonlinear, plateauing when the preceding RR interval exceeded 0.8 s, with a total r value of 0.45 (p <0.0001). All three regression parameter estimates were significant. In addition, LA strain was 1.1% higher (p = 0.02) in the VNS-treated animals, independent of preceding RR interval duration. The relationship between RRp/RRPpp and subsequent LA strain was weaker, with an r value of 0.28 (p = 0.001), and with only the LA strain_max_ term in the equation reaching statistical significance. However, VNS-treated animals again had higher LA strain (by 2.2%, p = 0.002) independently of preceding RRp/RRpp ratio. (Fig 6).

LA strain may be also influenced by LV diastolic function, specifically by the increase of LV filling pressures that could act as “afterload” to LA emptying during diastole. Table 1 summarizes the basic LV hemodynamics during the terminal study. While the VNS group showed borderline superior LV systolic pressure, and peak positive and negative derivatives, neither mean LV diastolic pressure or LV end-diastolic pressure was significantly increased in either group during AF following induction of HF.

## Discussion

In this paper, we explored how 6 months of pacing-induced and maintained AF, developed in the underlying presence of HF, affects LA size and function in a chronic dog model. In line with our prior study, [3] we found that, with animals in normal sinus rhythm, LA strain decreases and LA volume increases with the development of HF. With subsequent AF induction, LA strain decreased even further. The reduction in LA strain was attenuated in the dogs in which the ventricular response rate was slowed via VNS. In other words, VNS was beneficial in decreasing the negative impact of AF. However, after an initial dramatic increase in LA volume after the development of HF, neither subsequent AF induction nor VNS affected LA size. Finally, and somewhat surprisingly, once AF developed, no further changes were observed in either LA strain or LA volume throughout the follow-up period.

Prior studies have shown that sympathetic hyperinnervation of the left atrium that follows pathologic insults (e.g. rapid atrial pacing or myocardial infarction), facilitates AF induction [11] [12]. AF, once present, is associated with further increasing sympathetic stimulation both experimentally [13] and clinically [14]. While this makes VNS an attractive strategy to rectify autonomic imbalance and prevent AF, it appears that acute impact of VNS on atria during NSR depends on the stimulation level. VNS increases AF inducibility during high level stimulation [15], but opposite occurs with low-level stimulation [16]. On the other hand, chronic VNS delivery simultaneously with rapid atrial pacing does decrease AF inducibility and sympathetic nerve sprouting.[12]. These data fit well with our previous findings where chronic delivery of VNS simultaneously with rapid ventricular pacing decreased left atrial fibrosis and inflammation. [3]

In our study, VNS was started only after development of HF by rapid ventricular pacing. In other words, sympathetic hyperinnervation and atrial fibrosis have likely been present at the start of VNS, decreasing the magnitude of potential preventive benefit of VNS. Still, we have observed a smaller decrease in LA strain, once AF was present, in VNS treated group. After AF induction, LA strain decreased by ~ 60% in control animals, but by only ~30% in animals treated by VNS, with this difference between groups present throughout 6-months follow up. It is well documented that LA strain reflects LA fibrosis [17] [18]. However, it is likely that LA strain can be influenced by other factors, including rate and irregularity of RR intervals, and loading conditions.

Our data confirm that RR interval variability affects LA strain. This is expected, as long RR interval is associated with a long LV filling period [19], more thorough left atrial emptying, and smaller LA size at the beginning of the next cardiac cycle. Long preceding RR interval also leads to longer duration of next LV systole [20, 21] [22] and, as LA fills during LV systole, larger LA size. Importantly, VNS treated animals had higher LA strain even after controlling for preceding RR interval duration. We observed a similar impact of VNS on LA strain after accounting for the impact of the ratio between preceding and pre-preceding RR intervals (RRp/RRpp), a parameter of heart rate irregularity and a surrogate for LV contractility [23] [10]. In sum, LA strain is variable during AF, this variability is influenced by preceding RR interval duration, and VNS has beneficial effect on LA strains even after accounting for its effects on RR interval duration. In addition, similar hemodynamics between groups was observed at the end of the experiment (Table 1). These findings support possibility of lower LA atrial fibrosis as a plausible explanation for higher LA strains in the VNS group, which could be a potential target for future studies.

We have previously shown that HF initiated by tachycardia-induced cardiomyopathy in dogs leads to increased LA size.[24] Thus, the absence of further LA dilatation during 6 months of AF maintenance seems puzzling. A likely explanation is that the dramatic increase in LA size and atrial fibrosis induced by HF [25] masks any further dilatation following AF induction.

Of note, VNS appeared to attenuate the worsening of LA strain early (i.e. within two weeks) after the onset of AF, but without further impact detected throughout the follow up period. A rapid improvement in LA function (within first 2 to 4 weeks of VNS treatment) was expected, as it was previously demonstrated in dog and rat models of LA and/or left ventricular dysfunction [3, 26, 27]. However, the cause for the absence of further improvement in response to VNS is unclear. One possible mechanism is vagal nerve desensitization during continued electrical stimulation [28, 29]. However, further studies are needed to clarify this phenomenon.

### Clinical Implications

Our findings suggest that chronic VNS may improve LA function in AF. However, implementing VNS as performed in our study is highly invasive. On the other hand, recent developments in non-invasive VNS [16] may make it a useful, minimally invasive adjunct to other treatment options.

Given the increasing incidence of AF and greater use of LA strain assessment, it is important to understand the impact of AF on LA structure and function. We show that the presence of AF decreased LA strain by half, even with little change in average RR duration. We also show that LA strain is dependent on the preceding RR intervals, reaching a plateau for RR interval durations of ≥0.40s. This is consistent with our prior work with LV diastolic function parameters in AF [19], which demonstrated a similar dependency on the preceding RR interval. Given that the average baseline RR interval in our dogs was 0.54s, this implies that stable LA strain estimate in AF can be obtained as long as preceding RR interval is ≥75% of a predicted resting RR interval.

### Limitations

While we have explored the impact of AF that develops during underlying HF, we did not assess the impact of AF without preceding induction of HF, or the impact of induction of HF once AF develops. We also did not assess the correlation of the changes in LA strain with directly measured changes in LV function. However, we previously showed a very strong correlation between RR/RRpp ratio and various ventricular contractility measures, including LV end-systolic elastance, preload adjusted maximum power and dP/dt max. Other parameters, such as beat-to-beat changes in LV volumes, EF, or LV strain are challenging to quantify during AF using echocardiography, and thus would be inferior to using the simpler RRp/Rrpp ratio. We have not measured LA pressure directly. Finally, we were unable to perform any morphometric or histologic tissue analyses, due to technical factors.

In conclusion, this study documents the impact of AF development in the setting of HF on LA function and size. LA strain decreases further with the initiation of AF, with little impact of AF on an already increased LA size. Both LA size and function remained unchanged throughout a 6 month follow up period. Finally, LA strain shows a dependence on the duration of preceding RR intervals. Further studies are needed to corroborate our findings, and to assess its applicability and relevance in the clinical setting.

## Supporting Information

S1 Table Raw echocardiography data in individual animals obtained at baseline, after induction of heart failure (HF) and during subsequent follow-up with animals in atrial fibrillation (AF). LA: left atrial; LV: left ventricular; RRp/RRpp: ratio between preceding and pre-preceding RR intervals. 4c and 2c code for apical 4 chamber and 2 chamber echocardiographic views.
(XLSX)

S2 Table Raw hemodynamic data obtained at sacrifice. Max LVP: maximum left ventricular pressure; LV EDP: left ventricular end-diastolic pressure; LV Mean DP: mean diastolic left ventricular pressure; Max(Min) dLVP/dt: Maximal (minimal) first time derivative of left ventricular pressure; Tau: time constant of isovolumic pressure decay.
(XLSX)
